# Microplastic contamination and ecological risk assessment in two tree frog species (*Hyla orientalis* and *Hyla savignyi*) across Türkiye

**DOI:** 10.1007/s10653-026-03037-7

**Published:** 2026-02-08

**Authors:** Cantekin Dursun, Nagihan Demirci, Nurhayat Özdemir, Serkan Gül

**Affiliations:** 1 Department of Biology, Faculty of Arts and Sciences, Recep Tayyip Erdoğan University, 53100 Rize, Turkey; 2 Department of Biology, Faculty of Science, Karadeniz Technical University, 61080 Trabzon, Turkey

**Keywords:** Amphibians, Anura, Digestion, Frog, GITs, Pollution

## Abstract

This study was conducted to investigate the presence of microplastics (MPs) in individuals of *Hyla orientalis* and *Hyla savignyi*, two tree frog species naturally distributed in Türkiye, to determine the qualitative and quantitative distribution of these particles in their gastrointestinal tracts (GITs) and to analyze their morphological (color, shape, size) and chemical (polymer type) properties in detail. A total of 276 individuals were examined within the scope of the research, 76 of which belonged to *H. orientalis* and 200 to *H. savignyi*. A total of 192 microplastic particles were detected in their GITs, and the average size of these particles was determined to be 206.56 ± 12.88 µm. The most common microplastic type was PET (67.20%), its shape was fiber (76.00%), and its color was navy blue (25.50%). The highest proportion of microplastic-containing individuals was observed in *H. savignyi* (56.50%), and microplastic was found in only 11.84% of *H. orientalis* individuals. No statistically significant difference was found between the two species in terms of polymer type, microplastic shape, and color (*p* > 0.05). Data obtained from 24 different provinces across Türkiye indicate that microplastic contamination has a wide geographical distribution. The highest microplastic amount was recorded from Hatay-Hassa (44 pieces), followed by Kilis and Bitlis provinces. Significant differences were found between provinces in terms of color, shape, and polymer type (*p* < 0.001). These findings suggest that microplastic pollution is widespread in terrestrial vertebrates and may vary among species and geographic regions, suggesting that amphibians may be important bioindicators for monitoring ecosystem health.

**Supplementary Information:**

The online version contains supplementary material available at 10.1007/s10653-026-03037-7.

## Introduction

MPs, defined as plastic particles smaller than 5 mm in size, have become one of the most widespread and alarming components of modern environmental pollution (Hou & Rao, 2022). These pollutants are released directly into the environment from primary production processes and are formed through the physical, chemical, or biological degradation of larger plastic waste (Burgos-Aceves et al., 2022). MPs are an increasing concern in aquatic environments because of their ecotoxicological risks. Their ingestion by various organisms can deplete energy reserves, and they can accumulate within organisms through the food chain, becoming more concentrated over time. This accumulation leads to significant issues in the digestive, excretory, reproductive, and growth systems of affected organisms (Bakir et al., 2014). Furthermore, due to their relatively large surface area and hydrophobic nature, MPs readily adhere to various materials, including heavy metals (Akdoğan & Güven, 2019). The increasing detection of MPs in terrestrial, marine, and freshwater systems necessitate comprehensive investigation of the effects of these particles on biological systems (Rahman et al., 2024). While most studies on microplastic pollution have focused on marine organisms (Aytan et al., 2025; Onay et al., 2023, 2025; Şentürk Koca et al., 2023), limited information is still available on organisms living in freshwater habitats especially amphibians (Boyero et al., 2020; Najibzadeh et al., 2024). Amphibians are highly susceptible to environmental stressors due to their dual life cycles (aquatic and terrestrial stages) and semi-permeable skin (Bosch et al., 2021; Hu et al., 2022). Furthermore, the larval stage of many species is directly dependent on the aquatic environment, increasing the risk of exposure to MPs during this period (Kolenda et al., 2020; Teampanpong & Duengkae, 2024). Chemical pollutants, in particular, play a significant role in the global loss of amphibian biodiversity (Hayes et al., 2010). These substances can directly result in mortality or cause severe morphological, physiological, and reproductive disruptions. Given that amphibian embryos and larvae typically develop in freshwater habitats, pollutants in these environments negatively impact their physical and sexual development. Despite their ecological importance and the threats, they face, amphibians have historically been underrepresented in environmental research (Bókony et al., 2020). Furthermore, despite this sensitivity, the effects of MP pollution on amphibians have often been overlooked (Balestrieri et al., 2022).

Recent studies have shown that MPs can accumulate in the gastrointestinal tract of amphibians, causing tissue damage, increased oxidative stress, behavioral disorders, and growth retardation (Dmitrowicz et al., 2025; Najibzadeh et al., 2025; Park & Do, 2025). For example, it was experimentally demonstrated that MP exposure negatively affects growth, feeding, and survival rates in larvae (Boyero et al., 2020). Similarly, studies in South American and European species have reported that MPs accumulate in target tissues such as liver, intestine, and muscle (Balestrieri et al., 2022; e Souza Ferreira et al., 2025; Szkudlarek et al., 2024a). In addition, the detection of numerous microplastic particles settled in the digestive system of amphibians living in landscapes with intense agricultural activities indicates that the habitat-related effects of these pollutants should also be taken into consideration (Szkudlarek et al., 2023). MPs are reported to have effects not only on individual levels but also on ecosystem functions. For example, MPs attached to the periphyton layer can indirectly stress larvae by affecting their primary food source and disrupting the balance of food webs (Manríquez Guzmán et al., 2023; Shetu et al., 2023). This suggests that amphibians may also be source of MPs to terrestrial environments through trophic transfer (Buss et al., 2021; Dursun et al., 2023). The physical properties and chemical components of MPs are key parameters determining their environmental distribution and biological effects (Morais et al., 2024; Szkudlarek et al., 2024a). Therefore, in MP research, it is important not only to detect their presence but also to analyze their relationships with environmental matrices (Bosch et al., 2021; Kaya et al., 2025).

This study aimed (1) to investigate the presence of MPs in individuals of *H. orientalis* Bedriaga, 1890, and *H. savignyi* Audouin, 1827, two tree frog species found in Türkiye, (2) to qualitatively and quantitatively assess the presence of microplastic particles in the digestive tracts of these individuals, and (3) to analyze in detail the morphological characteristics and chemical composition of these particles.

## Materials and methods

### Fieldwork

**Fig. 1:**
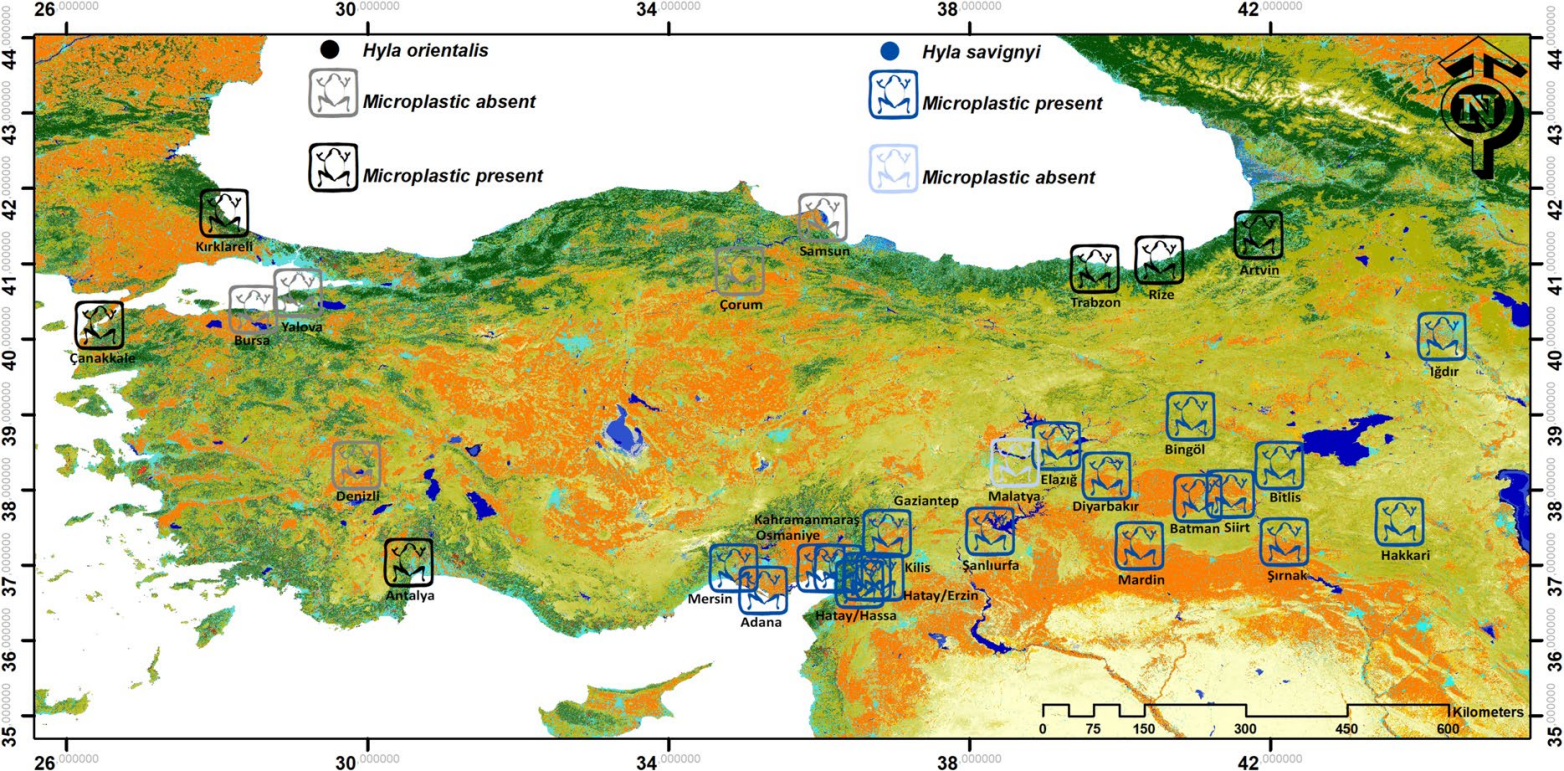
The sampling locations of the genus *Hyla* throughout Türkiye. Colors in the map present changes of land cover and land use between 2000 and 2020 (see Potapov et al. (2022) for color legend)

In the content of this study, a total of 30 different provinces were visited in Türkiye between 2023 and 2024 (Fig. 1). Fieldwork is done in the spring following breeding season. Captured frogs were euthanized with 250 mg/L MS222 (Tricaine methane sulfonate) and taken in glass jar containing 96% ethanol. The permission has been taken from Republic of Türkiye Recep Tayyip Erdoğan University Local Ethics Committee for Animal Experiments (Decision number: 2024/44).

### Microplastic characterization

MPs were isolated from GITs of adult frogs using a chemical digestion process involving a strong oxidizing agent—hydrogen peroxide (H_2_O_2_, 30% w/w). For digestion, weighted gastrointestinal tract were placed in glass tubes measuring 25 cm in length and 2.5 cm in diameter. These tubes were positioned within a batch reactor maintained at 65 °C. Each sample was treated with 5 mL of H_2_O_2_ solution per gram of tissue. Although the laboratory was not equipped with a HEPA-filtered laminar flow cabinet, strict contamination prevention measures were applied throughout all analytical procedures to minimize evaporation and contamination risk. Sample digestion and filtration were performed in a closed laboratory environment with restricted access. Cotton laboratory coats and single-use nitrile gloves were worn at all times, and only glass or stainless-steel equipment was used. All instruments, containers, and filter papers were thoroughly rinsed with distilled water prior to use. During digestion, samples were covered with watch glasses to prevent airborne contamination. Microplastic identification was further validated by FT-IR analysis, which reduced the risk of false-positive particle classification. Following digestion, the remaining content was subjected to vacuum filtration using Whatman Grade 4 qualitative filter paper (20–25 µm pore size). Filter papers were subsequently transferred to glass Petri dishes for microscopic evaluation.

**Fig. 2:**
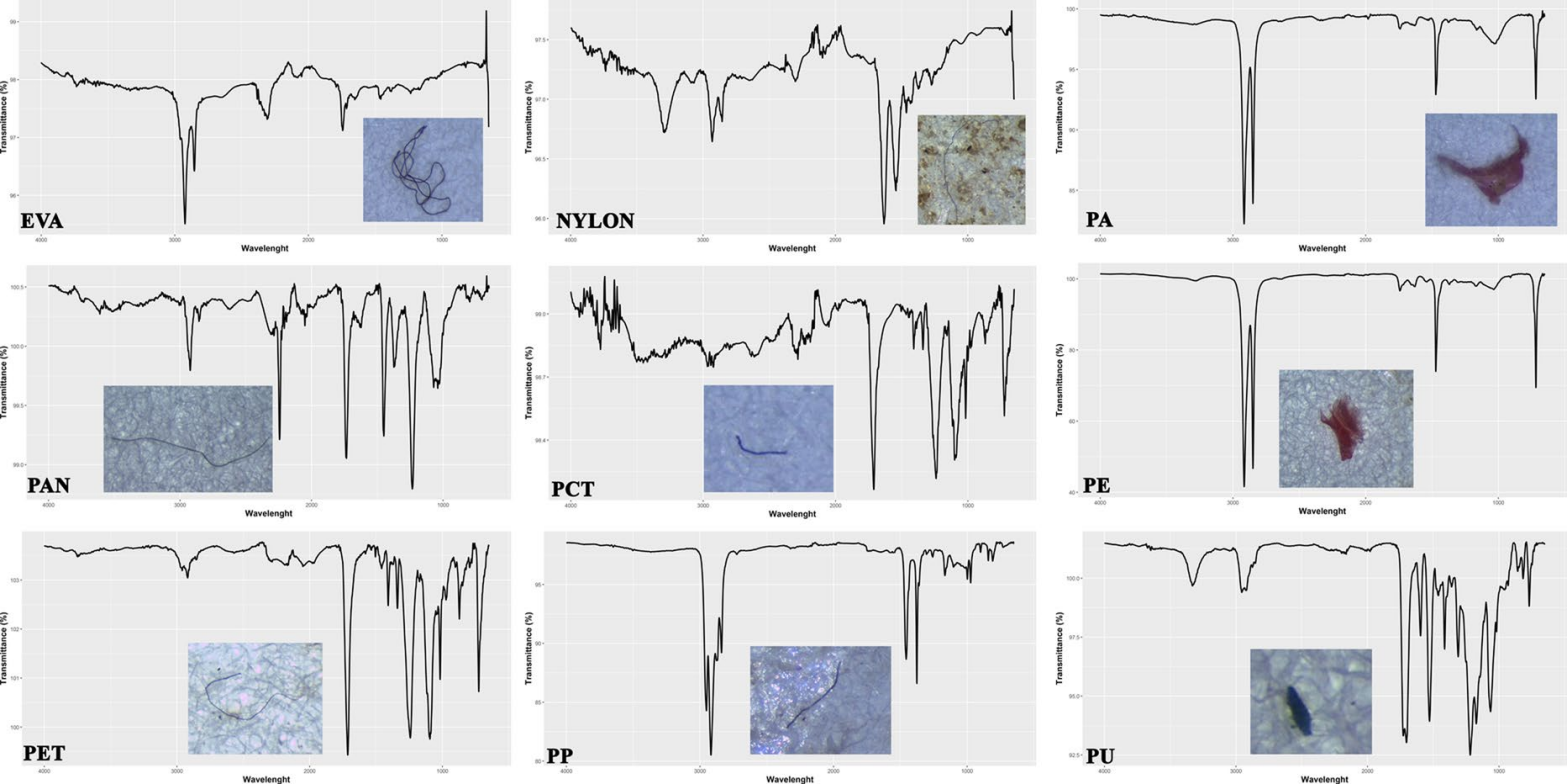
FT-IR spectra and match degrees of selected items obtained from gastrointestinal tracts

Suspected microplastic particles were detected using a Leica S6D® stereomicroscope (Wetzlar, Germany) based on observable traits such as color and morphology. For polymer identification, Fourier Transform Infrared Spectroscopy (FT-IR) was conducted using a PerkinElmer Spectrum 100 spectrometer equipped with an Attenuated Total Reflectance (ATR) accessory (PerkinElmer, Waltham, MA, USA). Spectral data were collected across a range of 4000–650 cm⁻^1^ with 12 scans per sample at a resolution of 2 cm⁻^1^. The resulting spectra were cross-checked with reference data from the PerkinElmer SEARCH Plus ATR Polymer library. Particles were classified as MPs if the spectral match exceeded a 70% similarity threshold (Fig. 2).

To calculate particle dimensions, images of isolated MPs were analysed using ImageJ software v1.46r (Aragón-Sánchez et al., 2017). A calibrated scale (1 mm at 4 × magnification) was used to measure the length of each particle. During the process, strict contamination control procedures were followed to minimize all risks. Inspectors used laboratory coats made of pure cotton and single-use nitrile gloves. All instruments and containers were composed of glass or stainless steel and were meticulously rinsed with distilled water before usage. Recovery efficiency was not experimentally assessed using spiked microplastic particles. However, the digestion protocol applied in this study (30% H_2_O_2_ at 65 °C) has been widely used in amphibian and freshwater microplastic research and is reported to preserve common polymer types such as PET, PE, PA, and PP. All detected particles were subsequently confirmed by FT-IR analysis, reducing the likelihood of analytical bias due to particle misidentification.

### Statistical analyses

Descriptive statistics, including means, standard errors, and ranges, were calculated separately for species, plastic type and provinces using *psych* package (Revelle, 2022). Data normality was assessed using the *olssr* package (Hebbali, 2020). Comparative analyses between the two species were conducted to evaluate differences in microplastic contamination characteristics. The Wilcoxon rank-sum test was employed to compare microplastic size and gastrointestinal tract weight (*p* < 0.05), while snout-vent length (SVL) differences were assessed using an independent samples t-test (*p* > 0.05) based on data distribution. Differences in categorical microplastic features (type, color, and shape) between species were analysed using Pearson’s chi-square test. To investigate differences in microplastic size among different color categories, the Kruskal–Wallis test was applied, followed by Dunn’s post hoc test for pairwise comparisons. A significance threshold of *p* < 0.05 was adopted for all tests. All statistical analyses were performed in *stats* package (R Core Team, 2025).

To demonstrate the relationship between MP shape and type, bubble plots were created using the *ggpubr* package (Kassambara, 2020), displaying the frequencies of different microplastic shapes and types across both *H. savignyi* and *H. orientalis* species. Correlations between quantitative variables were explored using pairwise plots generated with the *ggally* package (Schloerke et al., 2021). For qualitative microplastic characteristics, a radar plot was used to visualize the abundance frequency of different categories using the *fmsb* package (Nakazawa, 2024). To visualize the relationships between categorical variables, circle diagrams were constructed using the *circlize* package (Gu et al., 2014). These diagrams effectively matched species with various microplastic characteristics, creating links between categorical variables.

Stacked bar plots were employed to display the distribution of microplastic features across different provinces. Additionally, a heatmap was created to visualize the distribution of plastic types across provinces. A flow diagram was constructed comprising all dataset was shaped with *ggalluvial* package (Brunson, 2020). Lastly, the size distribution of MPs for both species was examined through histograms and a ridgeline plot to provide insights into the distribution of plastic sizes within each microplastic type. Visualization of the microplastic data was performed using *ggplot2* package (Wickham, 2016). All analyses were done in R Programming Language v4.5 (R Core Team, 2025).

### Polymer hazard index (PHI)

PHI was calculated for each species using the relative abundance of different polymer types and their associated hazard scores, as proposed by Lithner et al. (2011).

$$ {\mathrm{PHI}}\, = \,\sum (P_{n} \, \times \,S_{n} ). $$

where Pₙ is the percentage (%) of polymer n among all detected microplastic particles, and Sₙ is the hazard score assigned to polymer n. The polymer hazard scores (Table 1) were derived from the chemical hazard ranking of common polymers reported by Lithner et al. (2011). For each species, the relative percentage of each polymer type (Pₙ) was multiplied by its hazard score (Sₙ), and the results were summed to obtain the PHI value. PHI values were divided into five categories: low hazard (PHI < 150), medium hazard (150 < PHI < 300), considerable hazard (300 < PHI < 600), high hazard (600 < PHI < 1200), very high hazard (PHI > 1200).

**Table 1:** Hazard scores (Sₙ) assigned to major polymer types (after Lithner et al., 2011)

Polymer	Hazard score (Sₙ)	Reference/Note
Polyethylene (PE)	11	Lithner et al., 2011
Polypropylene (PP)	1	Lithner et al., 2011
Polyethylene terephthalate (PET)	4	Lithner et al., 2011
Polyamide (PA / Nylon)	47	Lithner et al., 2011
Polyurethane (PU)	7384	Lithner et al., 2011
Polyacrylonitrile (PAN)	10,599	Lithner et al., 2011
Ethylene–vinyl acetate (EVA)	9	Similar chemical structure, based on Lithner classification
PCT	4	Treated as PET-like due to chemical similarity

### Potential ecological risk index (PERI)

PERI incorporates both the hazard of each polymer type and the exposure level of the organism:

$$ {\mathrm{PERI}}\, = \,\sum (T_{i} \, \times \,CF). $$

$$ T_{i} \, = \,P_{n} \, \times \,S_{n} . $$

where Tᵢ represents the hazard contribution of polymer i, and CF is the contamination factor, defined as:

$$ CF = \frac{{\text{mean MP per contaminated individual}}}{{C_{0} }} $$

In this study, the reference concentration C₀ was set to 1 MP per contaminated individual. This normalization enables comparison between species by combining polymer hazard and exposure magnitude. PERI values were classified as minor (< 150), medium (150–300), high (300–600), danger (600–1200), and extreme danger (> 1200), respectively (Bae et al., 2025).

## Results

### Abundance and characterization of microplastics

In this study, 276 frogs were handled to monitoring microplastic contamination. The mean body size of *H. orientalis* samples was 36.82 ± 0.58 mm, mean gastrointestinal tract weight was 0.38 ± 0.03 g obtained from 76 individuals. For *H. savignyi*, mean body size was 36.51 ± 0.28 mm and mean gastrointestinal tract weight was 0.33 ± 0.02 g for 200 individuals. Descriptive table related to species and provinces are presented in Table 2.

**Table 2:** Descriptive table of dataset based on species and provinces (N: Number of individuals; MP: Microplastic; MP + : The number of individuals contaminated with MPs; SE: Standard error; Ho: *Hyla orientalis*; Hs: *Hyla savignyi*)

Species	N	Mean Body Length ± SE	Mean GIT Weight ± SE	N (MP +)	Number of MP	Mean MP Item	Mean MP Size ± SE	Range
*Hyla orientalis*	76	36.82 ± 0.58 mm	0.38 ± 0.03 g	9	9	1	293.59 ± 72.69 µm	64.16–590.81 µm
*Hyla savignyi*	200	36.51 ± 0.28 mm	0.33 ± 0.02 g	113	183	1.61	199.43 ± 12.90 µm	31.95–1026.53 µm

Province	N	Mean Body Length ± SE	Mean GIT Weight ± SE	N (MP +)	Number of MP	Mean MP Item	Mean MP Size ± SE	Range
Adana (Hs)	20	38.48 ± 0.78 mm	0.32 ± 0.03 g	3	2	0.66	363.73 ± 195.47 µm	168.26–559.20 µm
Antalya (Ho)	6	36.76 ± 1.22 mm	0.39 ± 0.06 g	1	1	1	116.79 ± 0.00 µm	–
Artvin (Ho)	8	36.53 ± 0.80 mm	0.32 ± 0.04 g	1	1	1	64.16 ± 0.00 µm	–
Batman (Hs)	11	36.74 ± 1.29 mm	0.30 ± 0.03 g	4	4	1	173.92 ± 58.51 µm	67.23–313.63 µm
Bingöl (Hs)	4	39.68 ± 2.17 mm	0.33 ± 0.08 g	1	1	1	594.25 ± 0.00 µm	–
Bitlis (Hs)	9	33.50 ± 1.50 mm	0.34 ± 0.04 g	7	24	3.42	220.43 ± 36.72 µm	58.54–845.43 µm
Bursa (Ho)	10	32.13 ± 0.62 mm	0.30 ± 0.03 g	0	0	0	–	–
Çanakkale (Ho)	2	44.05 ± 0.24 mm	1.11 ± 0.02 g	1	1	1	535.50 ± 0.00 µm	–
Çorum (Ho)	4	38.16 ± 0.45 mm	0.55 ± 0.07 g	0	0	0	–	–
Denizli (Ho)	5	35.68 ± 0.62 mm	0.30 ± 0.03 g	0	0	0	–	–
Diyarbakır (Hs)	10	37.70 ± 0.89 mm	0.28 ± 0.03 g	2	2	1	231.49 ± 175.45 µm	56.03–406.94 µm
Elazığ (Hs)	10	39.57 ± 1.50 mm	0.40 ± 0.04 g	4	5	1.25	154.02 ± 60.23 µm	50.96–362.93 µm
Gaziantep (Hs)	9	35.62 ± 1.17 mm	0.22 ± 0.04 g	6	11	1.83	214.99 ± 44.06 µm	58.42–487.00 µm
Hakkari (Hs)	3	36.08 ± 2.52 mm	0.10 ± 0.04 g	3	5	1.66	85.22 ± 21.98 µm	35.38–151.88 µm
Hatay-Hassa (Hs)	17	37.82 ± 0.86 mm	0.28 ± 0.02 g	10	44	4.4	187.83 ± 24.34 µm	33.08–976.04 µm
Hatay-Erzin (Hs)	11	38.52 ± 0.81 mm	0.30 ± 0.02 g	5	5	1	227.86 ± 60.68 µm	74.13–376.04 µm
Iğdır (Hs)	9	37.37 ± 0.68 mm	0.26 ± 0.01 g	3	4	1.33	211.49 ± 118.06 µm	71.89–564.11 µm
Kahramanmaraş (Hs)	10	34.51 ± 0.52 mm	0.25 ± 0.02 g	7	12	1.71	184.32 ± 40.73 µm	33.08–452.91 µm
Kilis (Hs)	10	36.37 ± 0.71 mm	0.27 ± 0.02 g	10	28	2.8	178.62 ± 34.88 µm	44.44–688.67 µm
Kırklareli (Ho)	10	36.05 ± 1.58 mm	0.32 ± 0.02 g	1	1	1	585.30 ± 0.00 µm	–
Malatya (Hs)	3	35.40 ± 1.58 mm	0.12 ± 0.03 g	0	0	0	–	–
Mardin (Hs)	8	36.50 ± 0.25 mm	0.97 ± 0.34 g	4	7	1.75	157.71 ± 36.20 µm	50.88–305.35 µm
Mersin (Hs)	15	33.20 ± 1.40 mm	0.36 ± 0.03 g	6	6	1	127.11 ± 29.44 µm	59.03–255.24 µm
Osmaniye (Hs)	14	34.63 ± 1.05 mm	0.20 ± 0.02 g	7	10	1.42	248.32 ± 61.60 µm	57.65–599.33 µm
Rize (Ho)	11	38.75 ± 1.25 mm	0.48 ± 0.09 g	2	3	1.5	193.84 ± 56.32 µm	130.68–306.19 µm
Samsun (Ho)	2	27.88 ± 0.99 mm	1.12 ± 0.07 g	0	0	0	–	–
Siirt (Hs)	7	38.87 ± 1.07 mm	0.34 ± 0.04 g	5	7	1.4	412.12 ± 128.79 µm	31.95–1026.53 µm
Şanlıurfa (Hs)	10	38.39 ± 1.07 mm	0.49 ± 0.08 g	5	5	1	104.14 ± 23.27 µm	40.57–156.19 µm
Şırnak (Hs)	10	38.78 ± 1.07 mm	0.24 ± 0.03 g	2	2	1	318.15 ± 37.36 µm	280.79–355.51 µm
Trabzon (Ho)	8	37.79 ± 0.91 mm	0.34 ± 0.04 g	1	1	1	590.81 ± 0.00 µm	–
Yalova (Ho)	10	40.27 ± 0.50 mm	0.26 ± 0.02 g	0	0	0	–	–

**Fig. 3:**
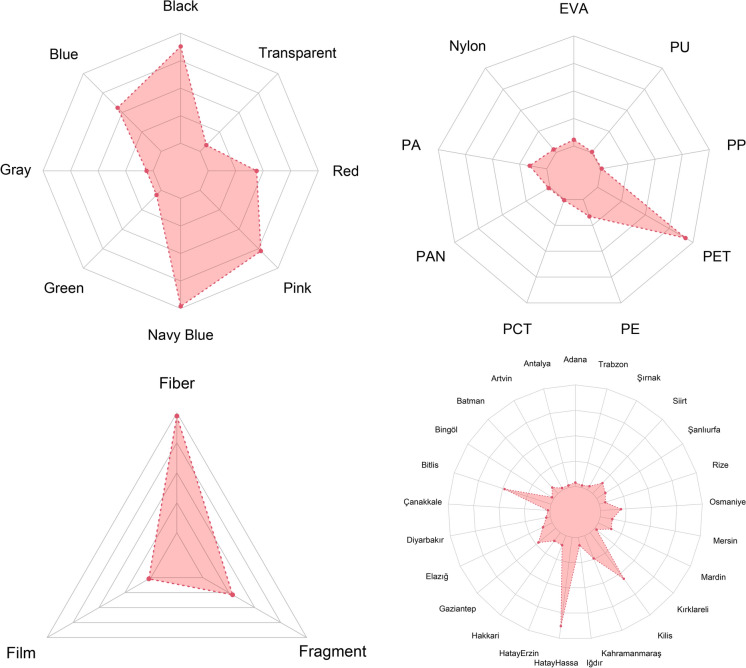
Radar plots of microplastic features based on their relative frequency with abundance and provinces. Areas above centre indicates a size increase with equal proportion

A total of 192 microplastic particles were identified in the gastrointestinal tracts of 123 frogs. The mean size of all microplastic items was found 206.56 ± 12.88 µm. The range was scaled between 31.95 and1026.53 µm. The mean SVL was 37.30 ± 0.26 mm ranged between 23.68 and 45.36 mm gastrointestinal tract weight was calculated 0.32 ± 0.02 g ranged from 0.05 to 2.29 g. There was no significant correlation between SVL– microplastic size (r = − 0.008; *p* > 0.05) and gastrointestinal tract weight– microplastic size (r = 0.03; *p* > 0.05), but a weak significant correlation between SVL and gastrointestinal tract weight (r = 0.14; *p* < 0.05). The correlogram including all quantitative variables was shown in Fig. S1. In total, 9 different microplastic types, 3 different microplastic shapes and 8 different microplastic color were unveiled from 25 different localities for all data set (Fig. 3).

**Fig. 4:**
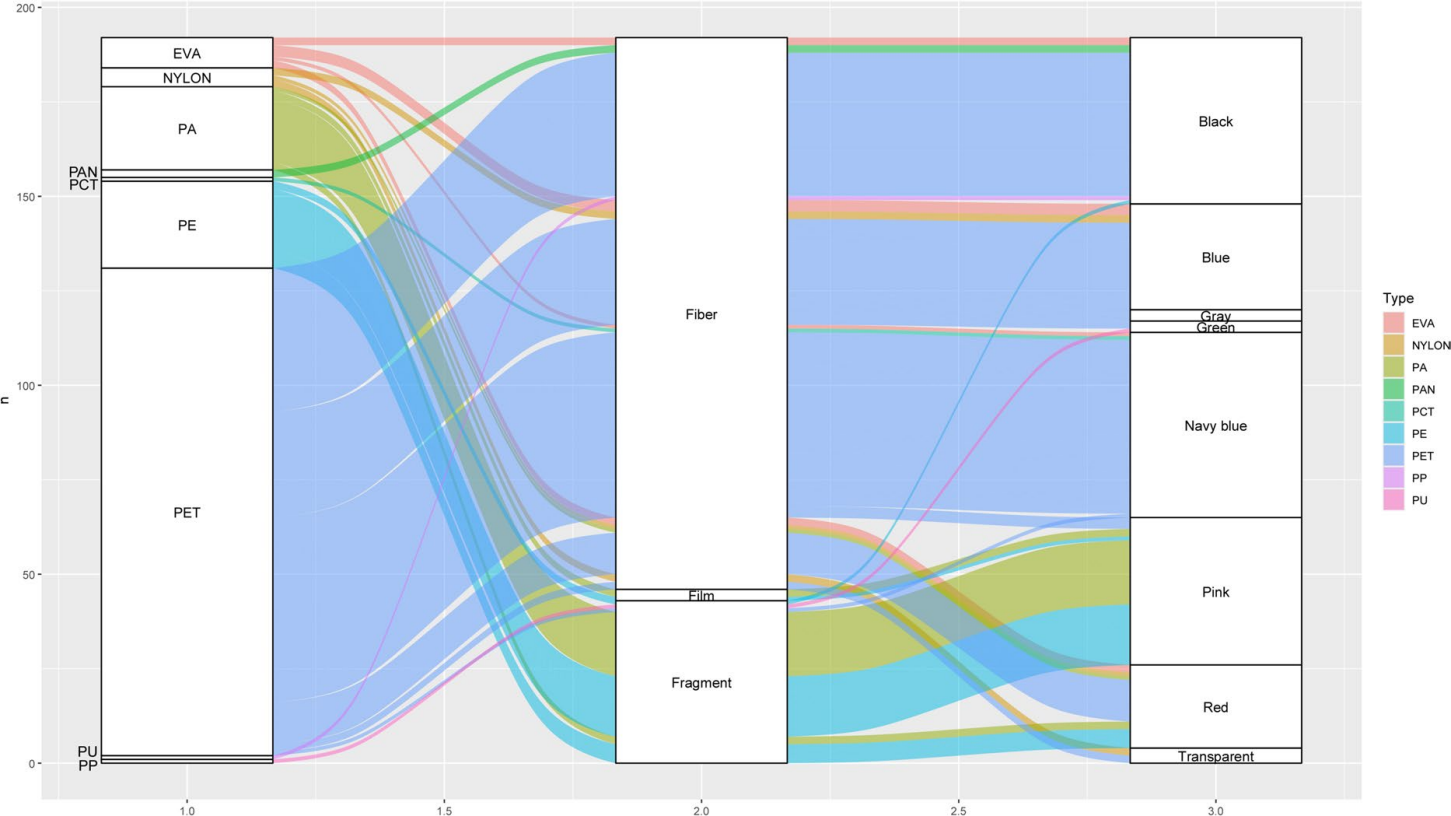
Alluvial diagram showing interaction strengths between nine polymers, three shapes and eight colors with subsequent distribution across observed microplastic items

**Fig. 5:**
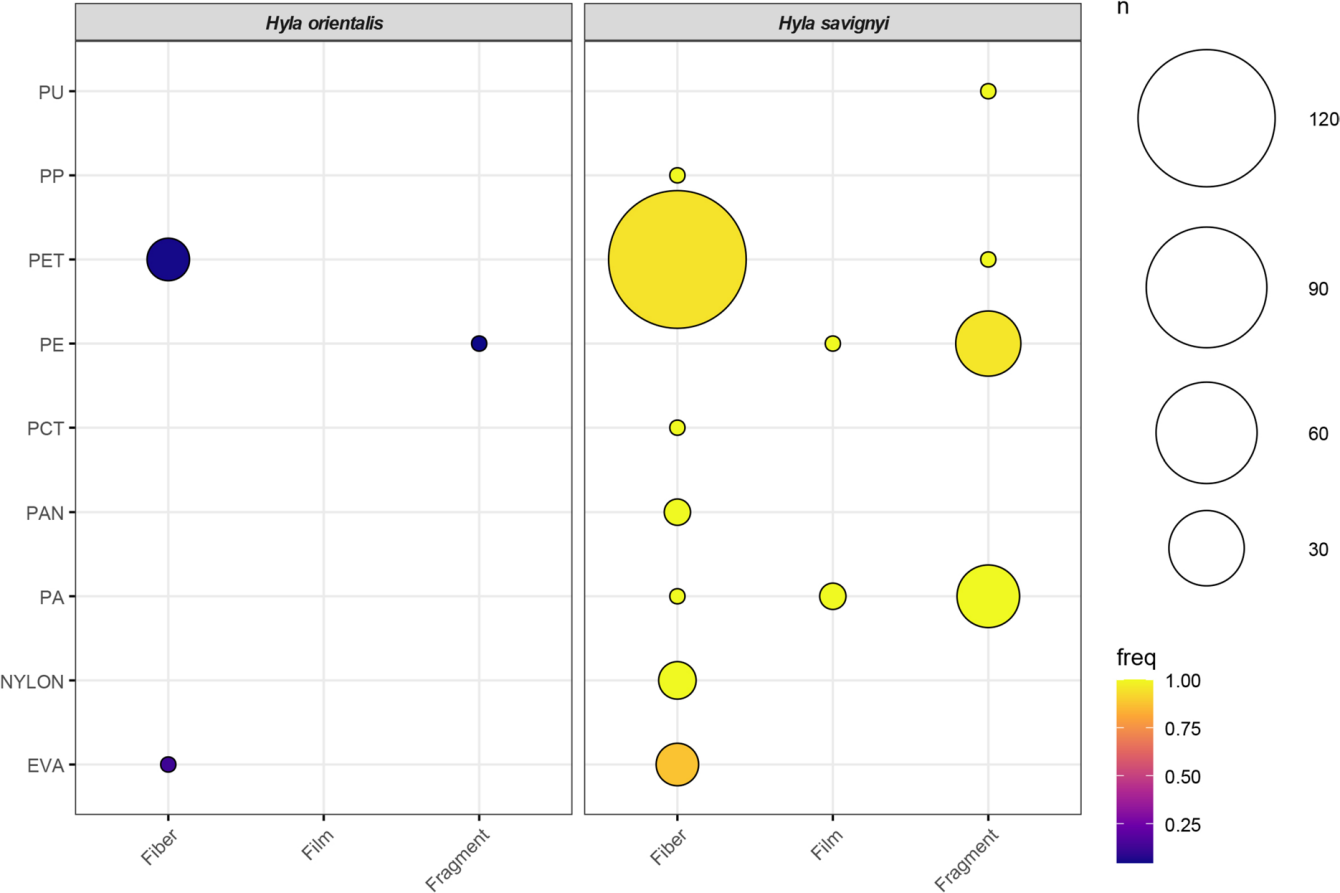
Bubble plot illustrating the relationship between plastic type and microplastic shape for each species. Bubble size corresponds to the number of items (n), while color intensity reflects the relative frequency (freq) of items by microplastic shape

The mostly observed microplastic characteristics were PET (n = 129; 67.20%), fiber (n = 146; 76.00%) and navy blue (n = 49; 25.50%). Most of fiber MPs were matched with PET (n = 128). However, there was no fiber items in PE (n = 23; 22 fragments, 1 film) and most of PA (21 of 22 items; 19 fragments, 2 films) and PP (1 fragment). These features were illustrated with links in Fig. 4. The largest mean microplastic size was found in PAN (297.97 ± 78.07 µm) while the smallest mean size was measured for PU (31.95 µm). Besides, the smallest item was measured in PU (31.95 µm) and the largest item was a PET (1026.53 µm). The corresponding color for these items were red and navy blue, respectively (Table 3). The largest mean microplastic size was in grey items (317.63 ± 25.56 µm), whereas the smallest mean size was in red items (148.95 ± 27.26 µm). Microplastic particle size demonstrated significant differences in terms of color categories (Kruskal–Wallis χ^2^ = 16.64; df = 7; *p* < 0.05; Fig. S2). Post hoc test indicated that the differences were observed between black and pink (*p* < 0.01), black and red (*p* < 0.05), grey and pink (*p* < 0.05), grey and red (*p* < 0.05), navy blue and pink (*p* < 0.05), transparent and pink (*p* < 0.05), transparent and red (*p* < 0.05). For observed MPs, 183 particles were found in 113 of 200 individuals of *H. savignyi* (56.50%) samples), while 9 particles were recovered from 9 of 76 individuals of *H. orientalis* (11.84%). In both species, polyethylene terephthalate (PET) was the most frequently detected polymer (Fig. 5).

**Table 3:** Descriptive table of dataset based on microplastic features (N: Number of individuals; SE: Standard error)

Plastic Type	N	Mean MP Size ± SE	Range	Plastic Color	N	Mean MP Size ± SE	Range	Plastic Shape	N	Mean MP Size ± SE	Range
EVA	8	153.83 ± 55.85 µm	41.23–535.5 µm	Black	44	220.22 ± 23.69 µm	41.23–702.01 µm	Fiber	146	219.33 ± 14.92 µm	33.08–1026.53 µm
NYLON	5	272.85 ± 51.98 µm	173.83–438.12 µm	Blue	28	191.56 ± 25.48 µm	33.20–594.25 µm	Fragment	43	142.98 ± 19.86 µm	31.95–594.96 µm
PA	22	156.22 ± 33.61 µm	35.38–688.67 µm	Gray	3	317.63 ± 25.56 µm	280.79–366.75 µm	Film	3	496.74 ± 187.92 µm	120.93–688.67 µm
PAN	2	297.97 ± 78.07 µm	219.9–376.04 µm	Green	3	197.40 ± 83.17 µm	31.95–294.96 µm				
PCT	1	40.57 ± 0.00 µm	–	Navy Blue	49	243.26 ± 33.57 µm	40.57–1026.53 µm				
PE	23	182.21 ± 37.99 µm	34.43–680.63 µm	Pink	39	171.56 ± 28.36 µm	35.38–688.67 µm				
PET	129	222.04 ± 16.27 µm	33.08–1026.53 µm	Red	22	148.95 ± 27.26 µm	33.08–535.5 µm				
PP	1	125.80 ± 0.00 µm	–	Transparent	4	293.56 ± 54.43 µm	200.00–417.62 µm				
PU	1	31.95 ± 0.00 µm	–

**Fig. 6:**
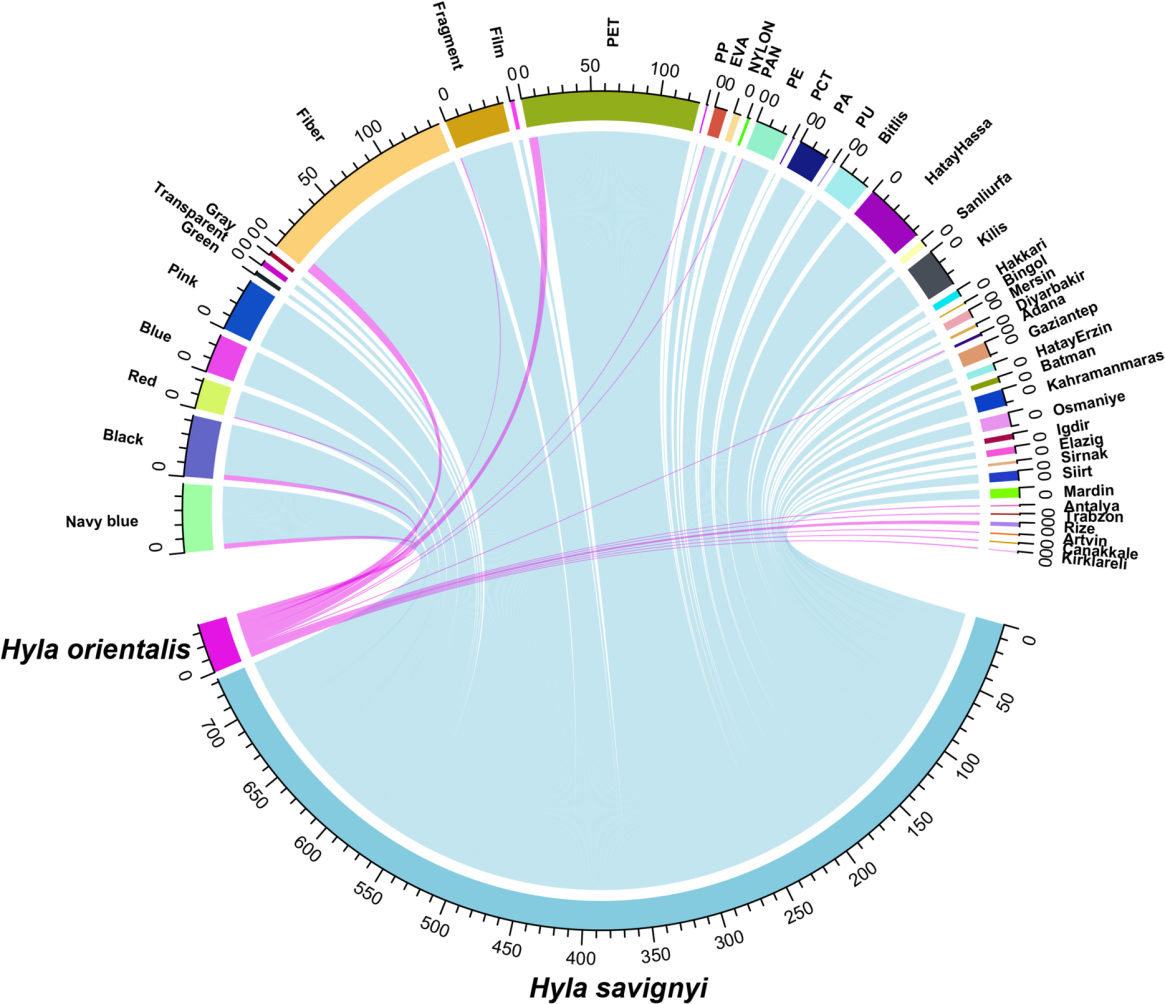
Chord diagram representing the complexity of the connections between species and categoric variables that pertain to characteristics of microplastics

**Fig. 7:**
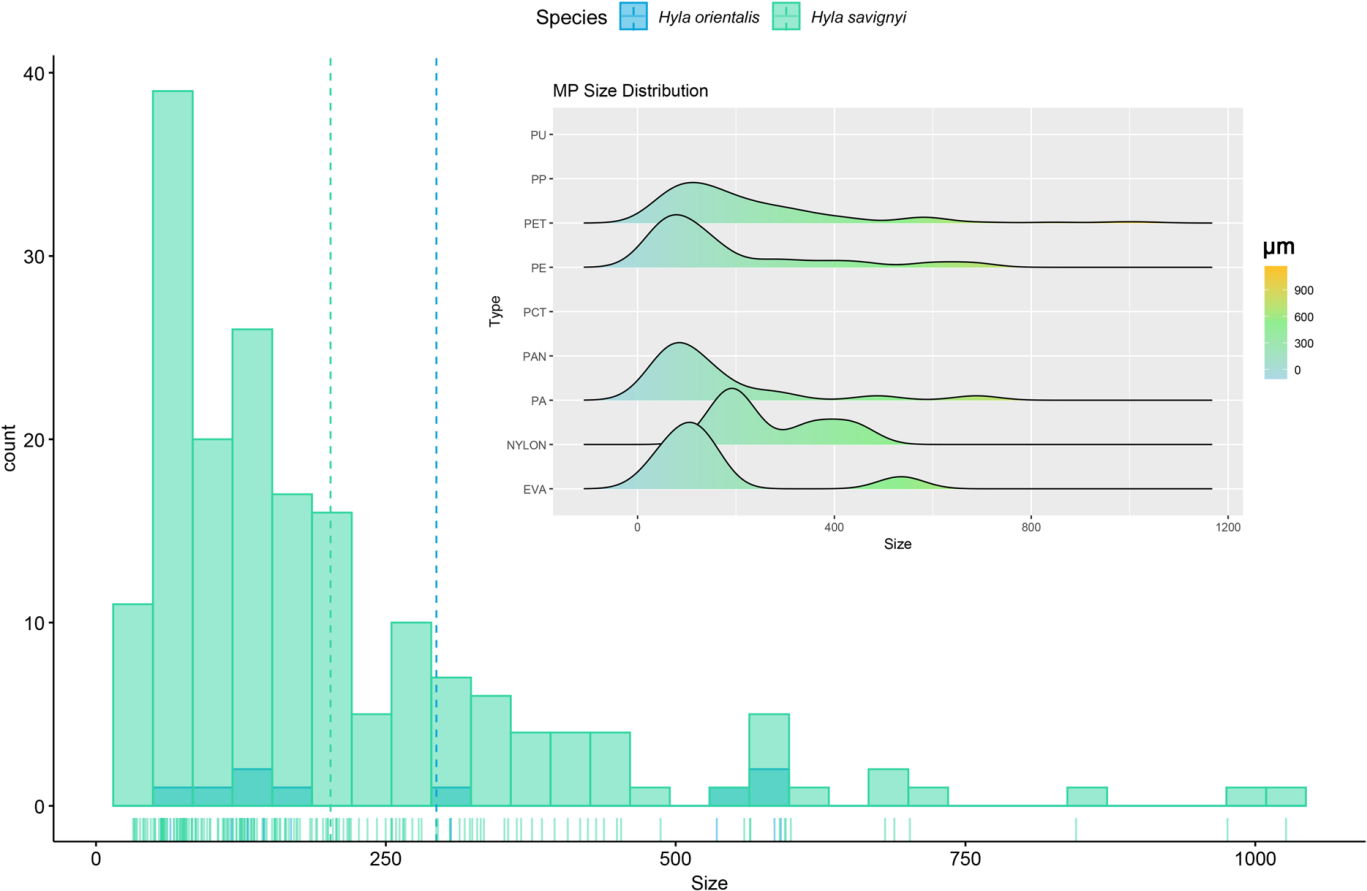
Size distribution of microplastics grouped by species. Dashed lines indicate the mean microplastic size for each species. The inset ridgeline plot displays the density distribution of microplastic sizes by plastic type. Plastic types with only a single observation (PU, PP, and PCT) are not represented in the distribution due to insufficient data

In *H. savignyi*, PET accounted for 121 items (66.10%), followed by polyethylene (PE; 21 items, 11.47%) and polyamide (PA; 10 items, 5.46%). Similarly, PET was the predominant polymer in *H. orientalis*, with 7 items (77.78%), followed by PE and ethylene–vinyl acetate (EVA), each represented by a single item (11.11%). Despite the higher total number of microplastic particles in *H. savignyi*, chi-square analysis indicated no statistically significant difference in polymer type composition between the two species (χ^2^ = 2.83; df = 8; *p* > 0.05). In terms of shape, fibers were the most common microplastic form in both species, comprising 138 particles (75.40%) in *H. savignyi* and 8 particles (88.89%) in *H. orientalis*. Fragments were the second most common, with 42 items (22.95%) in *H. savignyi* and one item (11.11%) in *H. orientalis*. Film particles were found only in *H. savignyi* (3 items, 1.63%). No significant difference in microplastic shape distribution was observed between the species (χ^2^ = 0.88; df = 2; *p* > 0.05). Regarding color, navy blue and black particles were the most frequently observed in both species (Fig. 6). In *H. savignyi*, 45 particles (24.59%) were navy blue and 40 (21.85%) were black. In *H. orientalis*, both colors were equally represented (4 items each; 44.44%). Other frequent colors in *H. savignyi* included pink (39 items, 21.31%) and blue (28 items, 15.05%), while a single red item (11.12%) was found in *H. orientalis*. However, no significant difference was found in microplastic color distribution between the species (χ^2^ = 7.02; df = 7; *p* > 0.05). Moreover, no significant interspecific differences were observed in snout–vent length (SVL; t = − 0.94; df = 8.8; *p* > 0.05), gastrointestinal tract weight (W = 794; *p* > 0.05), or mean MP particle size (W = 1073; *p* > 0.05; Fig. 7).

**Fig. 8:**
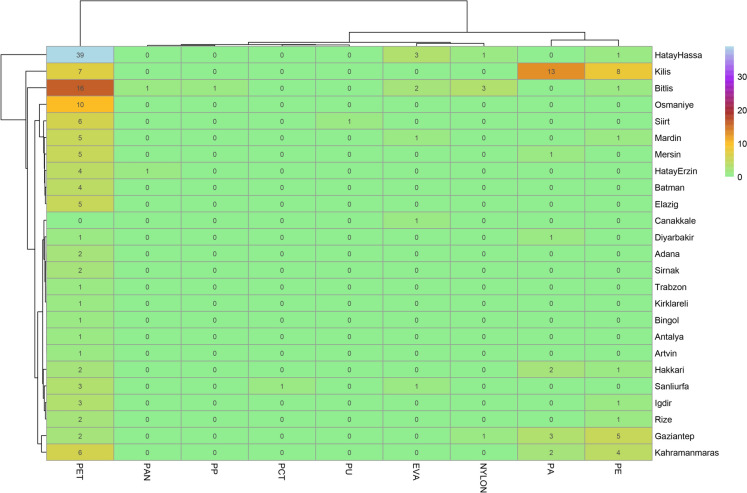
Heatmap illustrating the distribution of microplastic types across provinces. The color intensity reflects the abundance of microplastic type with their counts. The hierarchical clustering on both axes highlights patterns of similarity among provinces and microplastic types

Microplastic contamination was revealed in 24 different provinces. The highest number of microplastic items was recorded in Hatay–Hassa, accounting for 44 items (24.04% of the total items). This was followed by Kilis (28 items, 15.30%) and Bitlis (24 items, 13.11%). PET dominated observed plastics in Hatay-Hassa (39 items; 88.63%) and Bitlis (16 items; 66.67%), but PA was the most abundant type in Kilis (13 items; 46.42%). The minimum plastic size was measured 31.95 µm from Siirt and the maximum was 1026.53 µm from Siirt province. The highest number of microplastic items per individual was 4.4 from Hatay–Hassa followed by Bitlis with 3.42 items. The microplastic contaminated individual rate (N = 10) was 100% in Kilis, 77.77% in Bitlis (N = 7), 71.42% in Siirt, 70.00% in Kahramanmaraş, 66.67% in Gaziantep, and ranged between 50 and 58% in Şanlıurfa, Osmaniye, Mardin and Hatay–Hassa. The number of microplastic types in each province was presented as heatmap in Fig. 8. Statistically significant differences were observed among provinces in terms of microplastic color (χ^2^ = 251.06; df = 168; *p* < 0.001), shape (χ^2^ = 108.38; df = 48; *p* < 0.001), and polymer type (χ^2^ = 248.40; df = 192; *p* < 0.01). The number of MPs based on multiple categories with frequencies and stacked bars demonstrating percentage of microplastic characteristics for provinces were presented in Supplementary Material S1.

### Polymer hazard index (PHI)

The polymer hazard index (PHI) calculated for *H. savignyi* was 167,51 markedly higher than that of *H. orientalis* (10). This difference reflects both the higher abundance of microplastics in *H. savignyi* and the presence of polymers with relatively higher hazard scores, such as polyamide (Sₙ = 47), polyurethane (Sₙ = 7384), and polyacrylonitrile (Sₙ = 10,599). *H. savignyi* was found with medium hazard while *H. orientalis* was characterized with low hazard level. In contrast, MPs detected in *H. orientalis* were predominantly composed of low-hazard polymers (mainly PET and PE), resulting in a substantially lower PHI value. These results indicate that *H. savignyi* individuals are more strongly exposed to potentially hazardous polymer types than *H. orientalis*.

### Potential ecological risk index (PERI)

After integrating the contamination factor (CF) — the ratio of the mean number of MPs per contaminated individual to the reference value (C₀ = 1 MP/individual) — the calculated PERI values were 269,69 for *H. savignyi* and 10 for *H. orientalis*. *Hyla savignyi* was found with medium danger while *H. orientalis* was characterized with minor danger level The approximately 25-fold difference between species indicates that *H. savignyi* is subject to a considerably higher potential ecological risk from microplastic exposure. These findings are consistent with field-level contamination rates and polymer composition patterns observed in this study.

## Discussion

The presence of MPs in amphibians is an emerging environmental concern that remains understudied despite the vulnerability of these organisms to habitat degradation and pollution. Amphibians, due to their permeable skin, aquatic life stages, and position in the food web, are particularly susceptible to environmental pollutants such as MPs and they can be affected from various aspects such as physiological stress and intestinal homeostasis (Kyu-Park et al., 2024). However, it is known that only 0.4% of all extant amphibian species and 16% of all families were handled to investigate microplastic pollution (Rahman et al., 2024). In this aspect, this study was the first to detection of microplastic particles in the genus *Hyla* one of the genera in the family Hylidae distributing in the Old World (Dufresnes et al., 2020, Dufresnes and Litvinchuk, 2022). These frogs are generally described as semiaquatic and arboreal. Herein, GITs of two different species *H. savignyi* and *H. orientalis* across diverse ecological and geographic conditions added to growing evidence that MPs are infiltrating terrestrial and freshwater systems.

The findings indicate that the most abundant microplastic type was PET in both species, followed with PE and PA. Polyethylene terephthalate (PET) is a type of synthetic polymer commonly used in the production of plastic bottles, food containers, and synthetic fibers due to its durability, lightweight nature, and resistance to moisture. Due to extensively usage by people and improper disposal, the material is widespread in natural environments leading to environmental contamination. In many ecosystems, PET fragments can be ingested by animals either directly or through contaminated food sources (Provencher et al., 2022; Sawangproh & Paejaroen, 2025; Zhang et al., 2023). In amphibians, PET is also likely to encounter due to living in aquatic or semi-aquatic habitats. As example, e Souza-Ferreira et al. (2025) recorded first microplastic contamination for endemic Amazonian anuran species from Brazil and they noted that 11.10% isolated polymers were PET. Rahman et al. (2024) compiled all the literature to date on the presence of microplastics in amphibians and emphasized that PE, PET, and PS are the most common types of polymers in nature, as seen in our study. Hou and Rao (2022) said that plastics including phthalate esters such as PET are more possible to observe in amphibians because of the spread of urbanization and anthropogenic pollution with waste and debris. Karaoğlu and Gül (2020) inspected microplastic occurrence in two different species from Rize, Türkiye and they noted that PET had the highest frequency in characterized polymers. Besides, various studies handled aquatic or terrestrial amphibians supported the presence of PET in their gastrointestinal tracts (Dursun et al., 2023, 2024; Najibzadeh et al., 2025; Pastorino et al., 2022). The polyamide (PA) materials are commercially known as nylon and widely used in textiles and fishing gears. They can enter in natural habitats via wastewater discharge, surface runoff, or atmospheric deposition. Pastorino et al. (2022) monitored microplastics in *Rana temporaria* from Northwest Italy, and 60.00% of detected polymers were PA, and the rest was PET and PE. They also related the source of PA with clothes of tourists visiting the area. Szkudlarek et al. (2024b) examined microplastic diversity among 10 different amphibian species and determined 17 different MP types. However, PA was found only in *R. temporaria* and *Rana arvalis* larvae. From this aspect, PA was firstly reported in *Hyla* genus.

Considering the shape, fiber as the most prevalent shape (75.90%) then fragment and film, respectively. Fiber is a common pattern in monitoring studies handling terrestrial and freshwater environments, where synthetic textiles and packaging materials frequently break down into fibrous microplastics. Fragments emerge from disposal of larger and firm plastic materials, but they are irregular in terms of shape comparing to fibers. Films are also a secondary microplastics that more flexible and tend to be curled. Shetu et al. (2023) identified microplastics in 9 different frogs and toads from Bangladesh. They found that fibers were over 50.00% in 8 species and the second abundant shape was fragment. Kolenda et al. (2020) explained that microplastic ingestion was unveiled in pond-breeding amphibian tadpoles from Poland, and majority of particles were fibers (97%) followed by fragments (3%) as reported in other studies (Burger et al., 2024; Chuaynkern et al., 2024; Kuranova et al., 2024). Therefore, it can be thought that MPs contaminating amphibians show similar characteristics allowing to leak in gastrointestinal system.

In total, 8 different color was revealed in detected MPs dominated by blue, black and pink polymers. In the study of Szkudlarek et al. (2024b), blue and black items had higher proportion in 10 amphibian different species. They said that dominance of some colors in microplastic is not random due to their noticeability by predators or polymer origin. Besides, it was stated that blue plastics were characterized with faster degradation rate ineffectiveness against to ultraviolet radiation. Considering color-based differences in microplastic size were statistically significant in our study, this variation may stem from the differential sources and weathering behaviours of plastics, as well as potential visual selection or feeding biases in amphibians. Dursun et al. (2023) exhibited that blue and transparent colors were widespread in MPs for *Pelophylax ridibundus* along Türkiye, and black MPs were observed more than 50.00% of sampling stations. Najibzadeh et al. (2025) also pointed out that blue and black MPs had occurred in 4 different sampling site and two different species *Bufotes perrini* and *Pelophylax* sp. from Iran. Dursun et al. (2024) found similar patterns in *Neurergus* salamanders from Türkiye. Blue MPs especially arose from disposed plastic bottles made from PET which is most abundant plastic type in this study. Black MPs are more relevant with the materials such as vehicle tires and used outdoors or for heavy-duty use (pipes, containers, electronics, due to UV stabilizers or carbon black). Therefore, these MPs can be distributed along aquatic or terrestrial habitats with transportation or wastes and the findings in this study were in congruent with literature.

The mean microplastic size was approximately 204 µm, with a broad size range spanning from 24.03 µm to over 1 mm. Chuaynkern et al. (2024) sought for MPs in three different hylid frogs *Hylarana erythraea*, *Hoplobatrachus chinensis* and *Kaloula pulchra* and they reported the mean plastic size between 1453 and 1713 µm. Szkudlarek et al. (2024b) reported average fiber size was 840 μm, granule was 109 μm, flake was 95 μm, and fragment was 66 μm. In the study of Dursun et al. (2023), the mean microplastic size for *P. ridibundus* was calculated 735.95 μm. Pastorino et al. (2022) ranged microplastic size between 550.91 and 2355.51 μm for *R. temporaria* for blue and black items. Hu et al. (2022) inspected accumulation of MPs in different anuran larvae in China, and they said that plastics under 500 μm in earlier developmental stages for *Microhyla ornata*, *Microhyla heymonsi* and *Rana limnochari* species. Contrary to their observation, Burger et al. (2024) implied that larger *Amietia delalandii* (Common River frog) individuals have shorter microplastic fibers in their tissues comparing to smaller frogs. Given the presence of different observations in literature, it can be extracted that the shorter mean microplastic size is not directly linked with the smaller body size of *Hyla* frogs in this study. While microplastic size did not significantly correlate with SVL or gastrointestinal tract weight, the observed size variability could reflect differences in ingestion pathways or microplastic degradation states in the environment.

Out of 276 examined frogs, microplastic contamination was identified in 123 individuals (44.57%), with a markedly higher contamination rate in *H. savignyi* (57.00%) compared to *H. orientalis* (11.84%). The average microplastic in contaminated individuals was 1.61 and 1, respectively. Chuaynkern et al. (2024) counted average microplastic items per individual between 1.3 and 2.5 for hylid frogs. In the study of Kolenda et al. (2020), the average microplastic number was 1.33 (71 items from 53 tadpoles). Kuranova et al. (2024) estimated microplastic number as 3.5 per individual for *Rana amurensis* adults. The findings of Najibzadeh et al. (2025) presented that the microplastic number in digestive tract was 1.75 for *Bufotes perrini* and 2.67 for *Pelophylax* sp obtained from 4 different sampling areas. Dursun et al. (2023) found 3.26 items/individual for adult *P. ridibundus* and Szkudlarek et al. (2023) reported 2.76 items/individual from 663 microplastic contaminated tadpoles from 10 different amphibians. Considering the differences in literature and data in this study, this disparity may reflect specific characteristics in feeding habits, habitat preferences, or exposure levels across sampled regions. Interestingly, despite this high contamination rate, no significant differences were found in microplastic polymer, shape, or color distributions between *Hyla* species, suggesting that the MPs encountered are relatively consistent in origin and environmental availability.

MPs were detected in frog populations from 24 out of 30 surveyed provinces, highlighting the widespread geographic distribution of plastic pollution across the region. In *H. orientalis*, MPs were absent in 5 of the 11 provinces sampled, whereas *H. savignyi* exhibited microplastic contamination in 18 of 19 provinces, with Malatya as the sole exception. Among the sampled localities, Hatay–Hassa, Kilis, and Bitlis emerged as notable hotspots of contamination, characterized not only by the total number of microplastic particles detected but also by elevated MP counts per individual and distinct polymer-specific profiles.

Environmental and anthropogenic factors appear to play a pivotal role in shaping the prevalence and characteristics of MPs within these habitats. In Hatay–Hassa, frog specimens were collected from a pond situated in an area heavily impacted by debris and rubble following the catastrophic earthquake that struck Türkiye in 2023. The widespread distribution of structural wreckage likely contributed to the unintended release of MPs into both aquatic and terrestrial ecosystems. In Kilis, the collection site was a depression previously fed by spring waters, suggesting that hydrological transport may have facilitated the accumulation of plastic debris in this location. In Bitlis, the presence of beekeeping activities near the sampling site further underscores the role of direct human intervention, as discarded plastic materials associated with apicultural practices were frequently observed around the pond during the beekeeping season. Although polyethylene terephthalate (PET) was the most prevalent polymer across the study area, Kilis displayed a particularly anomalous profile with a 100% contamination rate dominated by polyamide (PA). This suggests the presence of a single, localized source contributing to the observed PA levels. Such regional disparities in microplastic prevalence and composition are likely reflective of differences in local plastic usage patterns, waste management efficacy, and environmental dynamics. Statistically significant variations in MP color, morphology, and polymer type across provinces further support the hypothesis that localized anthropogenic activities are major determinants of the environmental microplastic profiles encountered by amphibian species.

## Conclusion

This study provides the first comprehensive evidence of microplastic contamination in tree frogs of the genus *Hyla* across Türkiye. MPs were detected in both *H. savignyi* and *H. orientalis*, demonstrating that even arboreal and semi-aquatic amphibians are exposed to plastic pollution across a wide geographic range. The predominance of PET fibers and the widespread occurrence of MPs across multiple provinces indicate a strong influence of anthropogenic activities on amphibian habitats. Although no significant interspecific differences were observed in microplastic characteristics such as polymer type, shape, or color, *H. savignyi* exhibited a substantially higher contamination rate and ecological risk indices compared to *H. orientalis*. These findings suggest that species-specific ecological traits and habitat use may influence microplastic exposure intensity rather than the type of plastics encountered.

The application of polymer-based hazard (PHI) and potential ecological risk (PERI) indices highlights the value of integrating polymer composition and exposure levels when assessing microplastic-related risks in terrestrial vertebrates. Overall, this study underscores the suitability of amphibians as bioindicators of environmental microplastic pollution and emphasizes the need for future research incorporating experimental recovery assessments, tissue-level effects, and long-term ecological consequences of microplastic exposure in amphibian populations.

## Supplementary Information

Below is the link to the electronic supplementary material.

Supplementary file1 (PDF 780 KB)

## Data Availability

The authors declare that the data supporting the findings of this study are available within the paper and its Supplementary Information files.
