# Supplementary material for: Microplastic contamination and ecological risk assessment in two tree frog species (Hyla orientalis and Hyla savignyi) across Türkiye
Source: Environ Geochem Health. 2026 Feb 8;48(3):148. doi: 10.1007/s10653-026-03037-7 (PMC12883512; doi:10.1007/s10653-026-03037-7)
Supplement: Supplementary file 1 — Supplementary file1 (PDF 780 KB) [file 10653_2026_3037_MOESM1_ESM.pdf]

Table S1. Plastic distribution and number of items based on species, type and shape

```
# A tibble: 16 × 5
# Groups:   Species, Shape [5]
   Species         Shape    Type      n    freq
   <chr>           <fct>    <fct> <int>   <dbl>
 1 Hyla orientalis Fiber    EVA       1 0.125
 2 Hyla orientalis Fiber    PET       7 0.875
 3 Hyla orientalis Fragment PE        1 1
 4 Hyla savignyi   Fiber    EVA       7 0.0507
 5 Hyla savignyi   Fiber    NYLON     5 0.0362
 6 Hyla savignyi   Fiber    PA        1 0.00725
 7 Hyla savignyi   Fiber    PAN       2 0.0145
 8 Hyla savignyi   Fiber    PCT       1 0.00725
 9 Hyla savignyi   Fiber    PET     121 0.877
10 Hyla savignyi   Fiber    PP        1 0.00725
11 Hyla savignyi   Film     PA        2 0.667
12 Hyla savignyi   Film     PE        1 0.333
13 Hyla savignyi   Fragment PA       19 0.452
14 Hyla savignyi   Fragment PE       21 0.5
15 Hyla savignyi   Fragment PET       1 0.0238
16 Hyla savignyi   Fragment PU        1 0.0238
```

Table S2. Plastic distribution and number of items based on province, type and shape

```
 # A tibble: 55 × 5
# Groups:   Province, Shape [37]
   Province      Shape    Type      n   freq
   <fct>         <fct>    <fct> <int>  <dbl>
 1 Adana         Fiber    PET       2 1
 2 Antalya       Fiber    PET       1 1
 3 Artvin        Fiber    PET       1 1
 4 Batman        Fiber    PET       4 1
 5 Bingol        Fiber    PET       1 1
 6 Bitlis        Fiber    EVA       2 0.0870
 7 Bitlis        Fiber    NYLON     3 0.130
 8 Bitlis        Fiber    PAN       1 0.0435
 9 Bitlis        Fiber    PET      16 0.696
10 Bitlis        Fiber    PP        1 0.0435
11 Bitlis        Fragment PE        1 1
12 Canakkale     Fiber    EVA       1 1
13 Diyarbakir    Fiber    PET       1 1
14 Diyarbakir    Fragment PA        1 1
15 Elazig        Fiber    PET       5 1
16 Gaziantep     Fiber    NYLON     1 0.333
17 Gaziantep     Fiber    PET       2 0.667
18 Gaziantep     Fragment PA        3 0.375
19 Gaziantep     Fragment PE        5 0.625
20 Hakkari       Fiber    PET       2 1
21 Hakkari       Fragment PA        2 0.667
22 Hakkari       Fragment PE        1 0.333
23 HatayErzin    Fiber    PAN       1 0.2
24 HatayErzin    Fiber    PET       4 0.8
25 HatayHassa    Fiber    EVA       3 0.0698
26 HatayHassa    Fiber    NYLON     1 0.0233
27 HatayHassa    Fiber    PET      39 0.907
28 HatayHassa    Fragment PE        1 1
```

29	Igdir	Fiber	PET	3	1
30	Igdir	Fragment	PE	1	1
31	Kahramanmaras	Fiber	PET	5	1
32	Kahramanmaras	Fragment	PA	2	0.286
33	Kahramanmaras	Fragment	PE	4	0.571
34	Kahramanmaras	Fragment	PET	1	0.143
35	Kilis	Fiber	PET	7	1
36	Kilis	Film	PA	2	0.667
37	Kilis	Film	PE	1	0.333
38	Kilis	Fragment	PA	11	0.611
39	Kilis	Fragment	PE	7	0.389
40	Kirklareli	Fiber	PET	1	1
41	Mardin	Fiber	EVA	1	0.167
42	Mardin	Fiber	PET	5	0.833
43	Mardin	Fragment	PE	1	1
44	Mersin	Fiber	PA	1	0.167
45	Mersin	Fiber	PET	5	0.833
46	Osmaniye	Fiber	PET	10	1
47	Rize	Fiber	PET	2	1
48	Rize	Fragment	PE	1	1
49	Sanliurfa	Fiber	EVA	1	0.2
50	Sanliurfa	Fiber	PCT	1	0.2
51	Sanliurfa	Fiber	PET	3	0.6
52	Siirt	Fiber	PET	6	1
53	Siirt	Fragment	PU	1	1
54	Sirnak	Fiber	PET	2	1
55	Trabzon	Fiber	PET	1	1

Table S3. Plastic distribution and number of items based on type and shape

A tibble: 13 × 4
Groups: Shape [3]

	Shape	Type	n	freq
	<fct>	<fct>	<int>	<dbl>
1	Fiber	EVA	8	0.0548
2	Fiber	NYLON	5	0.0342
3	Fiber	PA	1	0.00685
4	Fiber	PAN	2	0.0137
5	Fiber	PCT	1	0.00685
6	Fiber	PET	128	0.877
7	Fiber	PP	1	0.00685
8	Film	PA	2	0.667
9	Film	PE	1	0.333
10	Fragment	PA	19	0.442
11	Fragment	PE	22	0.512
12	Fragment	PET	1	0.0233
13	Fragment	PU	1	0.0233

Table S4. Plastic distribution and number of items based on species and colour

	Hyla orientalis	*Hyla savignyi*
Black	4	40
Blue	0	28
Gray	0	3
Green	0	3
Navy blue	4	45
Pink	0	39
Red	1	21
Transparent	0	4

Table S5. Plastic distribution and number of items based on species and shape

	Hyla orientalis	*Hyla savignyi*
Fiber	8	138
Film	0	3
Fragment	1	42

Table S6. Plastic distribution and number of items based on species and type

	Hyla orientalis	*Hyla savignyi*
EVA	1	7
NYLON	0	5
PA	0	22
PAN	0	2
PCT	0	1
PE	1	22
PET	7	122
PP	0	1
PU	0	1

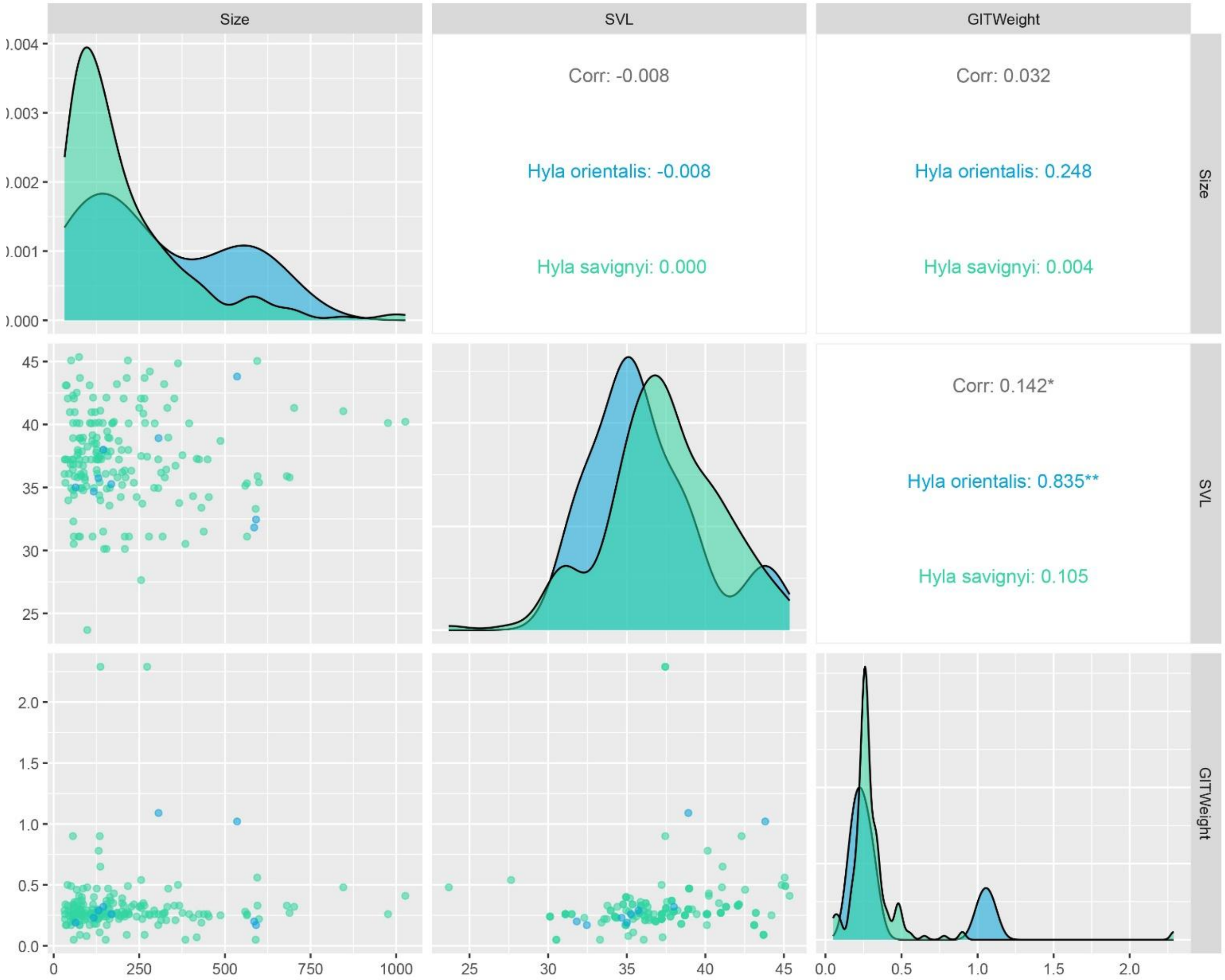

Figure S1. Pairs plot with correlation matrix. Corr values indicate the correlation coefficients (r). The significance level of correlation coefficients is represented with asterisk(s) (*, $p < 0.05$; **, $p < 0.01$).

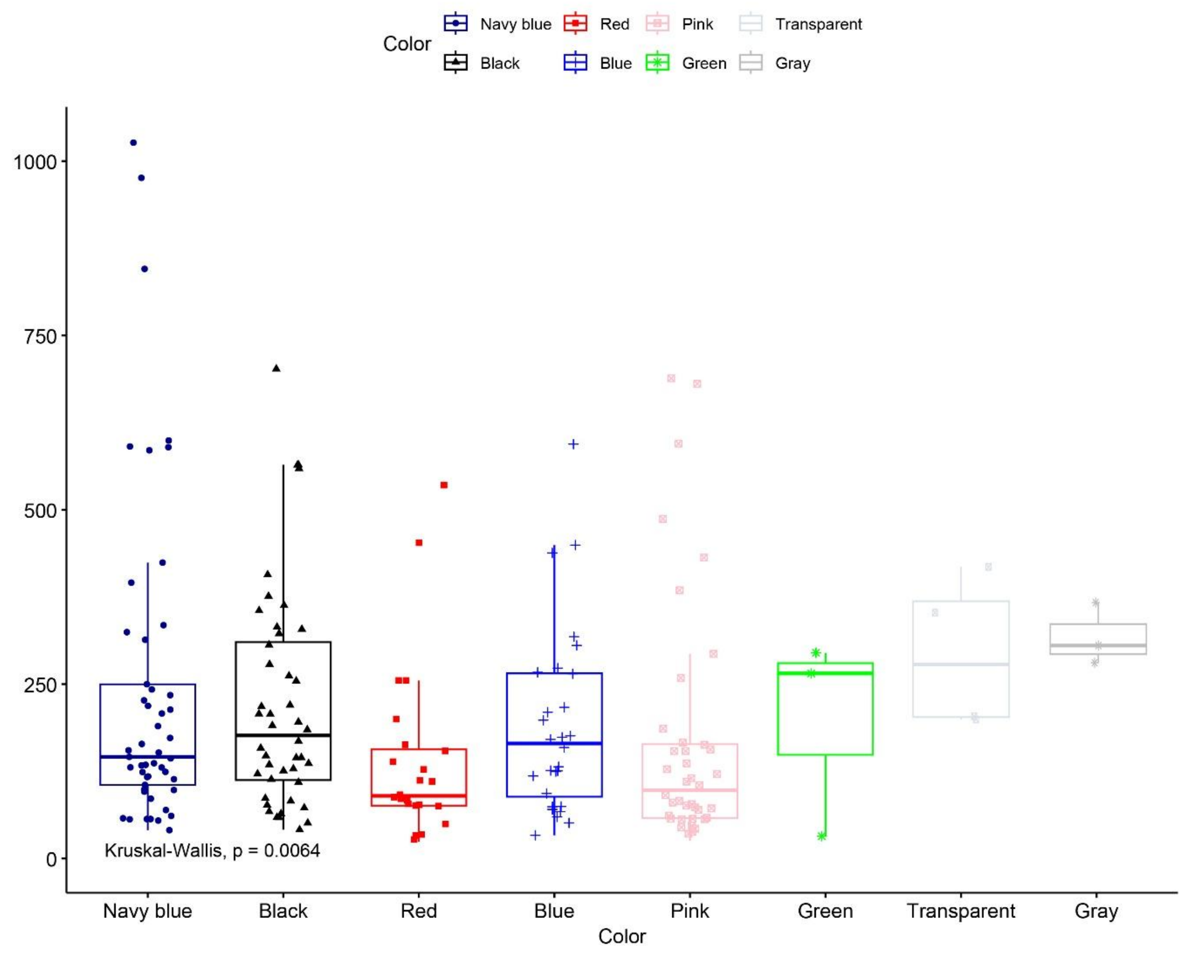

Figure S2. Boxplot of MP size distribution based on color categories. Line in box represents median value and lines on top and bottom represent quartiles. Each MP item were demonstrated with symbols corresponding size measurement on y axis.

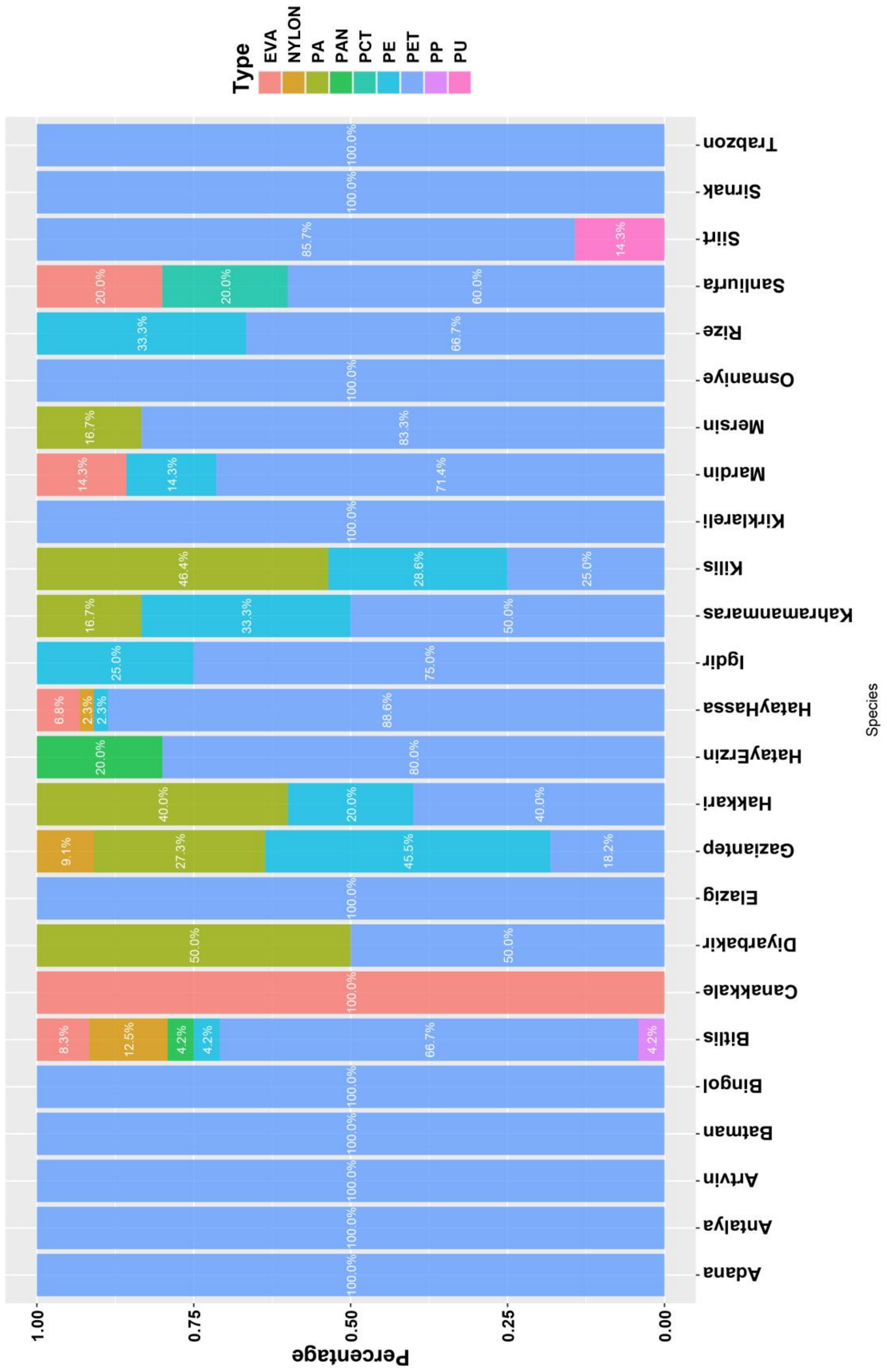

Figure S3. Stacked bar plots with relative percentage scores regarding abundance in each province for plastic type.

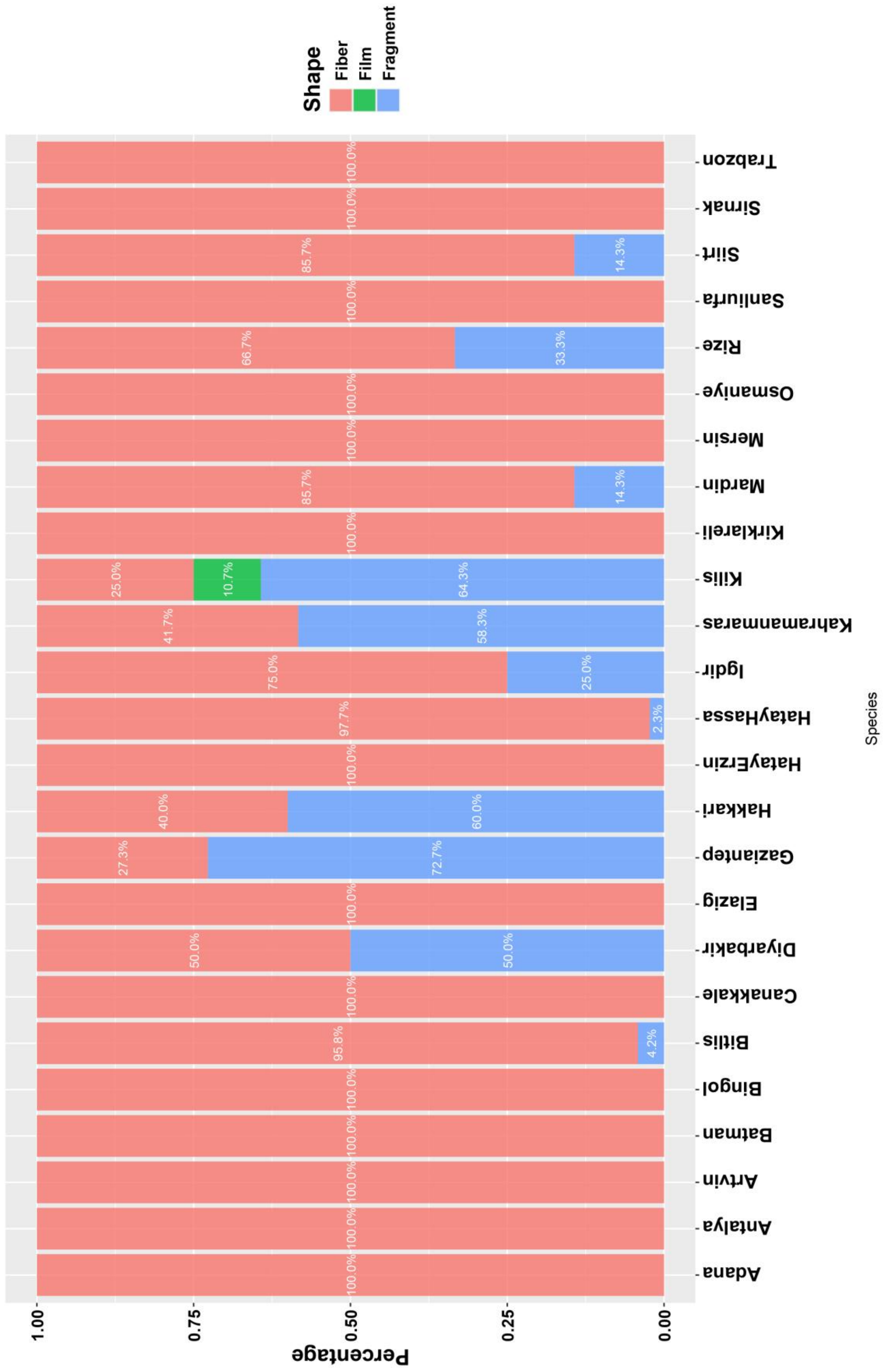

Figure S4. Stacked bar plots with relative percentage scores regarding abundance in each province for plastic shape.

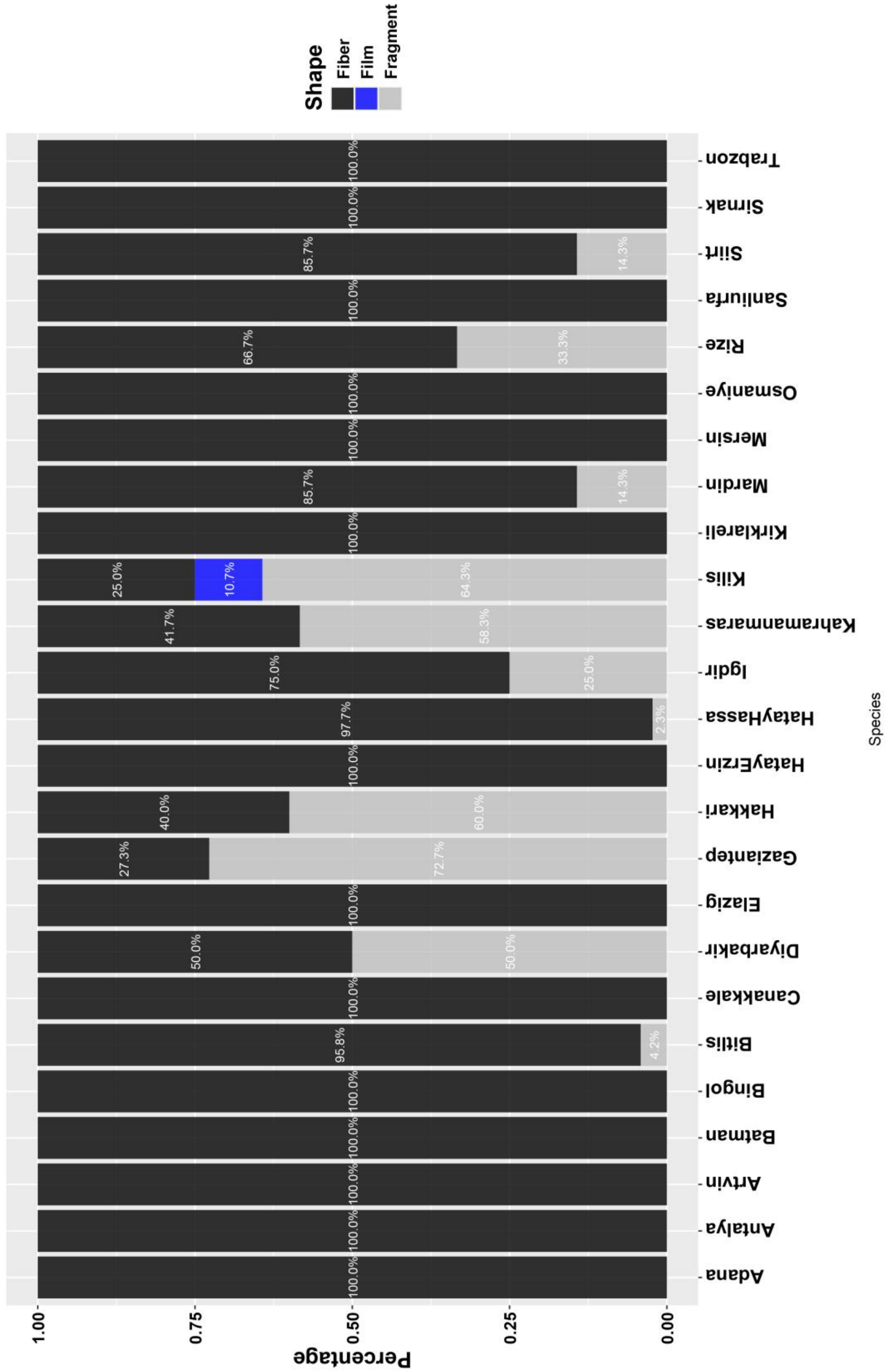

Figure S5. Stacked bar plots with relative percentage scores regarding abundance in each province for plastic type
